# Photodynamic Therapy in Dermatology

**DOI:** 10.3390/ijms27093960

**Published:** 2026-04-29

**Authors:** Antonio Di Guardo, Marco Virone, Umberto Gallo, Francesca Feresin, Antonio Ricupito, Roberta De Carolis, Vincenzo Coppolelli, Steven Paul Nisticò, Giovanni Pellacani, Carmen Cantisani

**Affiliations:** 1 UOC of Dermatology, Department of Medical and Cardiovascular Sciences, “Sapienza” University of Rome, 00161 Rome, Italy; vironem97@gmail.com (M.V.); umbertogallo2@gmail.com (U.G.); feresinfrancesca@gmail.com (F.F.); antonio.ricupito96@gmail.com (A.R.); robertadecarolis98@gmail.com (R.D.C.); vincenzo.coppolelli@uniroma1.it (V.C.); steven.nistico@uniroma1.it (S.P.N.); giovanni.pellacani@uniroma1.it (G.P.); 2 IRCCS Istituto Dermopatico dell’Immacolata (IDI-IRCCS), Dermatological Research Hospital, 00167 Rome, Italy

**Keywords:** photodynamic therapy, daylight photodynamic therapy, field cancerization, actini keratosis, basal cell carcinoma, infectious dermatology, photorejuvenation

## Abstract

Photodynamic therapy (PDT) is a minimally invasive treatment choice whose clinical success in dermatology relies on the interaction between a photosensitizer, light of an appropriate wavelength, and tissue oxygen, leading to reactive oxygen species generation and selective cytotoxicity. This narrative review summarizes contemporary mechanisms and clinical evidence supporting PDT across neoplastic, inflammatory, infectious, and esthetic indications. A comprehensive literature search included randomized trials when available, systematic reviews, meta-analyses, and guideline and consensus documents, complemented by mechanistic and translational studies relevant to clinical outcomes. In premalignant and neoplastic disease, strongest evidence supports field-directed PDT for actinic keratosis and high efficacy in Bowen’s disease, with favorable cosmetic outcomes and acceptable recurrence patterns. PDT plays a more selective role in basal cell carcinoma, particularly superficial and selected nodular lesions, while its routine use as monotherapy in squamous cell carcinoma remains limited by higher recurrence. Beyond oncology, PDT shows expanding utility in acne via sebomodulatory and immunomodulatory effects, and in infectious dermatoses through broad antimicrobial activity and biofilm disruption with low resistance potential. Cosmetic applications, including photorejuvenation, benefit from protocol tailoring and combination strategies that enhance penetration and remodeling. Overall, PDT is evolving into an adaptable therapeutic framework best positioned within mechanism-oriented, multimodal algorithms.

## 1. Introduction

**Figure 1:**
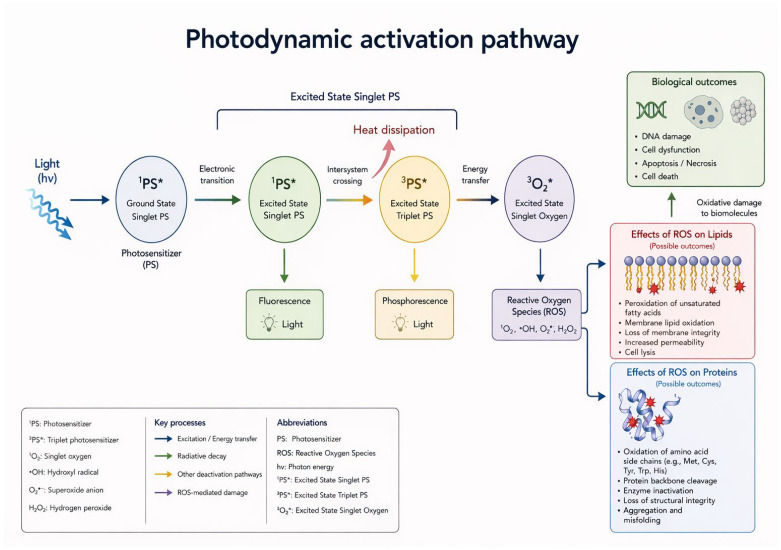
The image below illustrates the process by which PDT photosensitizers are activated, leading to the formation of free radicals.

Photodynamic therapy (PDT) occupies a unique position in dermatology, as it enables selective cytotoxicity against diseased skin while preserving surrounding structures, with proven efficacy in both oncological and non-oncological conditions. Its strengths lie in its efficacy, favorable outcomes, tolerability, and the possibility of repeating the treatment. Furthermore, it can be integrated with other therapeutic strategies. The conceptual origins of PDT can be traced back to ancient practices that combined plant-derived substances and sunlight for the treatment of skin disorders. However, a scientific framework was established in the early 20th century when Hermann von Tappeiner introduced the concept of “photodynamic action” through experiments using eosin as a photosensitizer [1]. Subsequent studies involving hematoporphyrin derivatives expanded the field. An important milestone was reached in 1990 with the introduction of 5-aminolevulinic acid (5-ALA) for topical use, which transformed PDT into a practical outpatient procedure and facilitated its widespread adoption in dermatology [2]. PDT has extended beyond dermatology and oncology into other medical specialties, including gynecology and urology, reflecting its versatility, selectivity, and favorable tolerability profile [3]. From a pathophysiological point of view, PDT relies on the interaction of three essential components, namely (i) a photosensitizer (PS), (ii) light of an appropriate wavelength, and (iii) molecular oxygen, together with the preferential accumulation or in situ generation of the active PS within diseased tissue [4]. Upon illumination within its absorption spectrum, the PS is excited from its ground state to an excited singlet state and subsequently transitions to a longer-lived excited triplet state (T1), which represents the key photoreactive intermediate. From this state, two main photochemical pathways are activated, leading to the generation of cytotoxic reactive oxygen species (ROS), particularly singlet oxygen, capable of damaging cellular structures such as membranes, mitochondria, lysosomes, proteins, and DNA, ultimately resulting in apoptosis and/or necrosis (Figure 1) [4,5,6,7].

In dermatology, modern PDT primarily uses topical precursors such as 5-ALA and methylaminolevulinate (MAL), which have largely overcome the unfavorable safety profiles of earlier photosensitizers [8]. Due to its low molecular weight, 5-ALA effectively penetrates the stratum corneum and is typically cleared within 24–48 h, thereby limiting prolonged photosensitivity [9]. MAL, a methyl ester of ALA, exhibits greater lipophilicity and may enhance tissue penetration, although it requires intracellular conversion back to ALA. In current clinical settings, conventional PDT (cPDT) typically uses artificial red-light-emitting diode (LED) sources that target the 630–635 nm absorption peak of PpIX to enhance tissue penetration. In contrast, daylight PDT (dlPDT) relies on natural sunlight, while artificial daylight PDT (adlPDT) employs broad-spectrum white light sources, with protocol modifications that may improve tolerability and feasibility in selected patient populations [10,11,12,13]. In this mechanistic context, PDT has become an established therapeutic option for a wide range of dermatological conditions, including actinic keratosis, superficial non-melanoma skin cancers, and certain inflammatory and infectious diseases, with growing interest in applications such as acne, photoaging, and viral warts. Contraindications include hypersensitivity to photosensitizer components or porphyrins, porphyria, systemic lupus erythematosus, and other photodermatoses. Patients must be adequately informed regarding post-treatment photoprotection, expected inflammatory reactions, and warning signs requiring medical evaluation [14].

This narrative review provides an up-to-date overview of PDT in dermatology, integrating its molecular mechanisms with the practical aspects of photosensitizers and illumination strategies, and examining established indications, evolving protocols, and emerging trends.

## 2. Results

In the following sections, we summarize the available clinical evidence regarding the efficacy and safety of PDT for a wide range of dermatological conditions, including neoplastic, inflammatory, infectious, and cosmetic indications. Attention is given to treatment response rates, recurrence patterns, and patient-centered clinical outcomes.

### 2.1. Neoplastic and Premalignant Dermatologic Indications

Across premalignant lesions, selected non-melanoma skin cancers, and high-risk clinical contexts, PDT represents a tissue-sparing and field-directed therapeutic option. Strong evidence supports its use in actinic keratosis (AK) and Bowen’s disease, while its role in squamous cell carcinoma (SCC) and basal cell carcinoma (BCC) is more selective or adjunctive. In addition, PDT is increasingly being investigated for its preventive potential in high-risk populations, such as immunosuppressed patients and individuals with genodermatoses.

#### 2.1.1. Actinic Keratosis

AKs represent the most common premalignant skin lesions. They are typically located on chronically sun-exposed areas, including the face, scalp, dorsal hands, and forearms. These lesions often occur in multiples within areas of field cancerization and may progress to squamous cell carcinoma (SCC) [14,15]. Clinical studies estimate that AK lesions may evolve into invasive SCC with an estimated annual progression rate of 0 to 0.075% per lesion [16]. Photodynamic therapy is particularly suitable for AK management because it allows treatment of large areas of affected skin, including entire fields of cancerization. Substantial clinical evidence supports the efficacy of PDL in AK treatment. Clinical trials report complete response (CR) rates of approximately 90% with MAL-PDT at three months [17,18] and from 84.5% to 87.6% with ALA-PDT at 6 months [19]. The effectiveness of PDT appears comparable to that of cryotherapy and topical 5-fluorouracil (5-FU). Two phase III studies comparing MAL-PDT with cryotherapy demonstrated that two PDT sessions achieved superior efficacy compared with a single freeze–thaw cryotherapy cycle, while one PDT session showed similar efficacy to two cryotherapy cycles [18,20]. Kurwa et al. reported a comparable reduction in mean lesion area between PDT-treated and fluorouracil-treated sites six months after treatment, whereas Smith et al. found similar short-term efficacy between PDT and fluorouracil one month after therapy [21,22]. Various treatment protocols and light sources have been proposed for PDT administration [11]. Conventional MAL-PDT (MAL-cPDT), which is recommended for Olsen grade I–II AKs and for field cancerization, has demonstrated high complete response rates [23]. However, practical considerations such as the availability of red-light sources and prolonged treatment times in the clinical setting may influence therapeutic decisions. Daylight-mediated PDT (MAL-dlPDT) has emerged as a convenient alternative. This approach has been associated with higher patient satisfaction in terms of perceived efficacy, reduced pain, and improved cosmetic outcomes [24]. Although limitations remain in the treatment of thicker AK lesions and variability in light exposure related to geographic and climatic factors, several studies indicate that indirect sunlight exposure can still produce effective results [25,26]. MAL-dlPDT requires only minimal environmental conditions, including temperatures above 10 °C and a minimum light dose of 8 J/cm^2^, to achieve adequate treatment outcomes. Furthermore, home-based protocols may allow patients greater autonomy in managing therapy [23]. In areas where natural daylight exposure is insufficient, artificial daylight PDT (MAL-adlPDT) represents a valid alternative with fewer logistical limitations. Some studies suggest that MAL-adlPDT may also provide longer-lasting remission in cases of extensive field cancerization. Overall, MAL-dlPDT represents a flexible and effective treatment strategy for AK, combining clinical efficacy with improved patient adherence and convenience [11]. In addition, a self-adhesive 5-ALA patch has been proposed to standardize topical drug delivery and simplify the PDT procedure. In a case series including 10 patients with a total of 40 AK lesions treated with a single conventional or daylight PDT session, all patients showed clinical responses with good-to-excellent tolerability and only mild local adverse effects [27]. Combination and sequential therapeutic strategies have also been investigated to enhance treatment efficacy in patients with multiple AKs. These approaches combine PDT with topical field-directed agents in order to improve lesion clearance while maintaining acceptable tolerability. Cantisani et al., for example, evaluated a sequential treatment protocol consisting of MAL daylight-PDT followed by topical diclofenac 3% combined with hyaluronic acid gel in patients with multiple AKs. Their findings support the feasibility of a staged therapeutic approach that combines procedure-based cytotoxic activity with topical anti-inflammatory field therapy [28]. Additional evidence of synergistic effects has been reported with the use of 5-fluorouracil pretreatment before ALA-PDT. In a randomized split-site study, pretreatment with 5-FU resulted in a greater reduction in AK counts compared with ALA-PDT alone at both six months (100% vs. 66.7%) and twelve months (100% vs. 82.6%) [29]. More recent trials have similarly evaluated short-course topical 5-FU followed by daylight PDT compared with daylight PDT alone [30]. In general, PDT is well tolerated in patients with AKs, although mild to moderate pain, erythema, and irritation may occur [31]. Cosmetic outcomes are particularly favorable, being rated as “good” or “excellent” by 96–98% of investigators [17,20]. Furthermore, cosmetic results are significantly superior to those obtained with cryotherapy after three months of follow-up. Therefore, PDT represents an especially suitable therapeutic option for actinic keratosis because it allows treatment of large areas of affected skin, provides excellent cosmetic outcomes, and is generally well tolerated.

#### 2.1.2. Bowen’s Disease

Bowen’s disease (BD) represents a form of squamous cell carcinoma in situ that typically manifests as an erythematous, eczematous plaque [32]. Approximately 3% of Bowen’s disease lesions eventually progress to invasive SCC [33]. Given the typical patient population and the frequent localization on areas with limited healing capacity, such as the lower extremities in elderly individuals with impaired vascular supply, therapeutic strategies must ensure both efficacy and good tolerability. Topical photodynamic therapy has been extensively investigated in this setting. Several open studies and four randomized clinical trials have demonstrated high efficacy of PDT in the treatment of Bowen’s disease [34,35,36]. Clinical studies using ALA-PDT have reported initial cure rates ranging from 88% to 100%, whereas MAL-PDT has shown an initial cure rate of approximately 93% [31,34,35,36]. At twelve months of follow-up, recurrence rates remained relatively low, estimated at about 15% with MAL-PDT and between 0% and 12% with ALA-PDT. Although long-term clearance data remain limited, the available evidence suggests outcomes comparable to those achieved with more commonly used therapies such as cryotherapy and topical 5-fluorouracil [37]. PDT is generally well tolerated and has demonstrated better tolerability compared with other treatment modalities. Pain represents the most common adverse effect, followed by erythema [32]. Cosmetic outcomes with MAL-PDT are also particularly favorable, with 89% of patients rating results as “good” or “excellent,” outperforming both cryotherapy and 5-FU [34].

#### 2.1.3. Squamous Cell Carcinoma

Squamous cell carcinomas (SCCs) are malignant skin tumors characterized by a relevant metastatic potential and, in some cases, by life-threatening clinical consequences. Standard therapeutic approaches for SCC include surgical excision, cryotherapy, topical treatments, and radiotherapy [38]. The use of photodynamic therapy in SCC has been investigated in a limited number of studies. Three open-label trials evaluated ALA-PDT in SCC lesions, reporting initial complete response rates ranging from 54% to 100% in superficial SCCs confined to the papillary dermis [39,40,41]. However, recurrence rates remain relatively high, reaching approximately 69%, and only 40% of non-superficial SCCs maintained clinical clearance after 36 months of follow-up. Evidence regarding the use of MAL-PDT in SCC treatment is still scarce. Although conventional PDT may show efficacy in selected superficial lesions, the relatively high recurrence rates limit its role in routine clinical management. For this reason, current evidence is insufficient to support the regular use of conventional PDT as monotherapy for SCC. In recent years, however, growing interest has emerged regarding the potential application of PDT in oral SCC and head and neck SCC, particularly with the development of targeted photosensitizers designed to improve tumor selectivity [42,43]. Moreover, PDT may be used as part of combination therapeutic strategies. A randomized controlled trial investigated MAL-PDT combined with fractional ablative laser treatment in patients with microinvasive SCC. The combined approach resulted in a complete response rate of 84.2% at three months, with a recurrence rate of 12.5%. In addition, cosmetic outcomes were considered excellent [43].

#### 2.1.4. Basal Cell Carcinoma

Basal cell carcinoma (BCC) frequently occurs in cosmetically sensitive areas, particularly on the face, and is also commonly diagnosed in relatively young patients. Photodynamic therapy represents a non-invasive treatment option for BCC, and substantial evidence supports its efficacy in the management of superficial basal cell carcinomas (sBCCs) [44]. Clinical trials have reported complete clearance rates at three months following MAL-PDT ranging from approximately 80% in difficult-to-treat BCCs to 97% in primary superficial BCCs, including cases with histologically confirmed clearance [45,46]. Other studies have reported complete response rates of 74% and >80% at 12 and 36 months, respectively, after MAL-PDT treatment [47,48]. Zou et al. in their metanalysis concluded that PDT is comparably effective to excision for treatment of BCC, but with increased risk of recurrence [49]. ALA-PDT has also been investigated for the treatment of superficial BCCs, demonstrating initial clearance rates between 92% and 97% [50]. In a randomized open-label prospective study, de Vijlder et al. reported a complete response rate of 88% at five years in superficial BCCs treated with ALA-PDT [51]. Recurrence rates following PDT appear generally comparable to those observed with other non-surgical therapies, although surgery remains superior in terms of long-term tumor control. In superficial BCCs measuring 1 cm or less, recurrence rates at 36 months were as low as 6%. In more difficult-to-treat superficial BCCs, recurrence rates after 36 months ranged between 15% and 31%, supporting the use of PDT as a valid therapeutic alternative in selected cases [46,47]. Cosmetic outcomes with MAL-PDT are particularly favorable. At three months, cosmetic results were rated as “good” or “excellent” by 89% of patients, compared with 50% of those treated with cryotherapy [52]. Similar findings have been reported in smaller randomized studies, including the work by Wang et al. [53]. In difficult-to-treat populations, MAL-PDT also demonstrated excellent cosmetic outcomes, with 94% of patients reporting “good” or “excellent” results after 24 months [46]. In nodular basal cell carcinoma (nBCC), several systemic photosensitizers, including porfimer, verteporfin, and mTHPC, have shown response rates ranging from 78% to 92% [54]. However, their use is limited by the systemic photosensitization they induce and their relatively low selectivity. Topical MAL has proven more effective in penetrating thicker nodular lesions. Clinical studies report complete response rates ranging from 73% to 94% when MAL-PDT is used for nBCC treatment [46,47,55,56,57]. Comparative studies with surgery indicate that MAL-PDT achieved a three-month response rate of approximately 91%, compared with 98% for surgical treatment [46]. ALA-PDT has also been evaluated in nodular BCC, although reported response rates are more variable, ranging from 61% to 92% [56]. The lower efficacy observed in some studies may be related to insufficient drug penetration and to variations in light delivery protocols [51]. Long-term outcomes remain encouraging. The five-year recurrence rate after MAL-PDT has been estimated at approximately 14% [56]. In difficult-to-treat nodular BCCs, recurrence rates between 48 and 60 months range from 18% to 30% [46,47]. Other studies have reported recurrence rates of 7% for thin nodular BCCs and 14% for thicker lesions after 35 months of follow-up [55]. For ALA-PDT, the recurrence rate at twelve months has been reported at approximately 12% [54]. Cosmetic outcomes in nodular BCC treated with PDT remain highly favorable, with 82% to 95% of patients reporting results rated as “good” or “excellent” [46,47,55,56,57]. In particular, cosmetic outcomes are generally superior to those achieved with cryotherapy or surgery, especially when lesions occur in cosmetically sensitive areas [54,56]. In pigmented basal cell carcinoma (pBCC), melanin present within stable protein complexes competes with the photosensitizer for photon absorption due to its broad absorption spectrum. This competition can reduce phototoxic effects and therefore limit treatment efficacy [58]. However, advances in highly active photosensitizers and in near-infrared light activation (700–900 nm) have expanded the potential role of PDT in pigmented lesions. Available evidence indicates that untreated pigmented BCCs generally show lower complete response rates to PDT [58]. Nevertheless, when superficial pigmentation is first removed through curettage or debridement, response rates improve significantly and may reach values comparable to those observed in other BCC subtypes, ranging from 50% to 100% [59,60]. A comparative overview of the clinical role, efficacy, and limitations of PDT across AK, BCC, and SCC is summarized in Table 1.

#### 2.1.5. NMSC Prevention in Immunosuppressed Patients and Patients with Gorlin Syndrome

PDT with ALA or MAL has attracted considerable interest as a preventive strategy for NMSCs and precancerous lesions in high-risk populations. These include immunocompromised patients who have undergone organ transplantation and those with Gorlin syndrome. Organ transplant recipients have a significantly increased risk of developing both basal cell carcinoma and squamous cell carcinoma. In this group, actinic keratoses often progress more rapidly to squamous cell carcinoma and are frequently associated with the development of multiple tumors and a more aggressive clinical course. Consequently, prevention strategies are particularly important in these patients. Preclinical studies conducted on mouse models have shown that repeated treatments with ALA-PDT or MAL-PDT can delay or even prevent the development of ultraviolet-induced skin tumors. In some experiments, no tumors developed in mice treated with ALA-PDT during a one-year observation period [61]. Although clinical data in humans are still limited, the initial results are encouraging. In a study of 27 transplant recipients, a single session of MAL-PDT significantly prolonged the median time to the development of a new actinic keratosis compared with untreated patients (9.6 months versus 6.8 months). Furthermore, a randomized multicenter study comparing contralateral treatment areas reported a significantly lower number of new actinic keratosis lesions after three months in areas treated with MAL-PDT compared to those receiving standard therapy (44 versus 80 lesions; *p* = 0.009) [62]. Further evidence supporting a preventive role for PDT has emerged from experimental studies using mouse models of Gorlin syndrome, as well as from clinical case reports involving patients with the syndrome. These observations suggest that PDT may exert a protective effect even in this specific genetic context. Overall, preliminary results indicate that PDT may help prevent or delay the development of non-melanoma skin cancers in high-risk populations. However, larger clinical trials and longer follow-up periods are needed to confirm the long-term efficacy, safety, and optimal treatment protocols for preventive PDT in these patients [51].

#### 2.1.6. Paget’s Disease

While surgical intervention has long served as the cornerstone of management for both mammary and extramammary Paget’s disease, its clinical utility is often hampered by significant long-term morbidity. This is particularly evident in large extramammary lesions, where radical excision frequently proves suboptimal. Patients undergoing surgery for vulvar Paget’s disease, for instance, often report persistent pruritus alongside profound functional and psychosexual impairment, challenges that underscore the urgent need for less invasive therapeutic strategies with more favorable side-effect profiles. In this landscape, PDT has emerged as a compelling alternative. Current evidence indicates that PDT can achieve complete clinical remission while effectively mitigating pruritus without inducing chronic adverse effects, even in the context of recurrent disease. A 2011 meta-analysis of 23 case reports and series, comprising 99 patients and 133 lesions, demonstrated a complete remission rate in 77 cases at one-year follow-up [63]. Subsequent studies in 2014 and 2018 have reinforced these observations, documenting sustained complete responses in diffuse or refractory cases with follow-up periods extending to 36 months [64]. The comparative efficacy of PDT versus surgery remains an area of active investigation. However, data from a randomized pilot study involving 13 patients are particularly promising; the study compared standard surgery against a combined approach of four ALA-PDT sessions followed by surgery. The results indicated a significantly lower local recurrence rate for the PDT combined with surgery group (9.1% at one year) compared to the group treated with surgery alone (25%), with a statistically significant difference (*p* = 0.0042) [65]. Despite these encouraging signals suggesting a transformative role for PDT in Paget’s disease, the current literature is still characterized by small-scale observations. Large-format, multicenter randomized controlled trials remain essential to validate these findings and establish definitive clinical guidelines.

#### 2.1.7. Primary Cutaneous Lymphoma

Primary cutaneous lymphomas (PCL) constitute a heterogeneous group of lymphoproliferative neoplasms that, by definition, involve only the skin at the time of diagnosis [66]. For patients with indolent disease limited to the skin, topical and skin-targeted interventions remain the preferred therapeutic strategy; within this therapeutic arsenal, PDT has emerged as a particularly promising modality. The clinical utility of PDT is particularly evident in localized and early-stage malignant tumors, including lymphomatoid papulosis (LyP), early-stage mycosis fungoides (MF), and certain cutaneous B-cell lymphomas (CBCL), with the marginal zone subtype showing remarkable sensitivity. The pathophysiological rationale for its use is based on a dual synergistic action: direct cytotoxicity mediated by RO and a robust immune-mediated response, characterized by the induction of immunogenic cell death. In clinical practice, PDT has demonstrated remarkable efficacy in the management of refractory LyP lesions, achieving durable local control. Similarly, in early-stage MF, the application of photosensitizers such as 5-ALA or MAL has been shown to induce both clinical and histopathological remission. Although the results obtained in CBCL cases are promising, the depth of skin infiltration sometimes poses an obstacle to light penetration and the diffusion of the photosensitizer, a limitation that is increasingly being overcome through the use of additional techniques, such as microneedling, aimed at improving therapeutic efficacy. Despite these encouraging observations, the current evidence base derives primarily from small-scale cohorts, and there remains a lack of direct comparative data against established standards for skin treatment. This gap in the literature prevents the definitive integration of PDT into standardized treatment algorithms. Consequently, there is an urgent need for large-scale randomized controlled clinical trials to validate long-term outcomes, optimize administration protocols, and refine the selection of photosensitizers for these specific oncological indications [67].

#### 2.1.8. Kaposi’s Sarcoma

Recent clinical studies have begun to evaluate the potential of PDT in the management of Kaposi’s sarcoma (KS), a multifocal vascular neoplasm caused primarily by human herpesvirus 8 (HHV-8) infection. Data from a targeted case series involving four patients treated with 16% MAL-PDT, using 630 nm red light irradiation after a three-hour incubation period, indicated substantial lesion regression or complete clinical resolution. These results were typically achieved within a median of 3–5 treatment sessions, yielding superior cosmetic outcomes along with negligible and self-limiting adverse effects [68]. In particular, the therapeutic response appeared to be influenced by both the patient’s age and the lesion’s morphology; greater efficacy was observed in younger cohorts and in superficial lesions, while older patients and those with non-HIV-associated KS showed more modest clinical improvements. Further strengthening the evidence in favor of systemic interventions, a case report involving a 79-year-old patient with nodular KS of the ankle demonstrated the efficacy of intravenous PDT using Photosens. After seven sessions over a six-month period, the patient achieved both clinical and histopathological remission [69]. A key technical aspect of this study was the integration of video-fluorescence diagnostics, which allowed for precise delineation of tumor margins and real-time monitoring of photobleaching, a physiological parameter closely correlated with the final therapeutic success. At the four-month follow-up, no recurrence was documented [69]. Although these preliminary data suggest that PDT, whether administered via topical MAL or systemic photosensitizers, offers a valid and safe alternative for cutaneous KS (particularly for patients for whom conventional therapies are contraindicated), the current clinical landscape remains fragmented. Consequently, robust, large-scale studies are essential to establish standardized protocols and optimize treatment parameters specifically tailored to the unique vascular architecture of these tumors.

#### 2.1.9. Melanoma

The clinical application of PDT in the management of melanoma has historically been limited by the tumor’s inherent resistance, which counterfaces from a multifaceted resistance profile. Optically, the high concentration of melanin constitutes a formidable barrier. By absorbing and scattering incident light, the pigment prevents the effective penetration of photons and the consequent activation of photosensitizers [70]. In addition to its role as an optical shield, melanin is a potent antioxidant and redox buffer, eliminating ROS that are fundamental to PDT-induced cytotoxicity. At the cellular level, therapeutic efficacy is further compromised by the presence of melanosomes, specialized organelles capable of sequestering photosensitizers, thereby reducing their bioavailability within critical subcellular compartments. This is exacerbated by the upregulation of ATP-binding cassette membrane transporters, such as ABCG2, which facilitate the active efflux of photosensitizers, keeping their intracellular concentrations below the threshold required for a lethal photodynamic effect. Furthermore, dysregulated apoptotic pathways often render melanoma cells less sensitive to programmed cell death signals typically triggered by photo-oxidative stress. To overcome these obstacles, current research is focusing on several innovative strategic approaches. One promising avenue involves the development of near-infrared (NIR) photosensitizers that operate in the 700–900 nm range; this shift to longer wavelengths improves tissue penetration while minimizing competitive absorption by melanin. In addition, additional depigmentation strategies, for example using tyrosinase inhibitors such as phenylthiourea or laser-assisted photobleaching, are being explored to temporarily light-sensitize the tumor microenvironment [70]. The integration of hyperthermia has also demonstrated synergistic potential and amplified oxidative damage when combined with PDT. Perhaps most significantly, the advent of in situ photoimmunotherapy (ISPI) and the concomitant administration of dendritic cell injections suggest that PDT can be harnessed to trigger a robust and sustained systemic immune response. When combined with efflux pump inhibitors or compounds designed to restore apoptotic sensitivity and modulate kinase activity, these combinatorial approaches offer the potential for a paradigm shift. Although melanoma has long been considered inherently unsuitable for PDT, these emerging findings provide a compelling rationale for its future incorporation into the treatment algorithm, even for advanced or metastatic forms [70].

### 2.2. Inflammatory and Infectious Indications

Beyond its established oncologic applications, photodynamic therapy has progressively expanded into inflammatory and infectious dermatology, where its combined sebomodulatory, immunomodulatory, and antimicrobial properties support a growing range of clinical indications.

#### 2.2.1. Acne Vulgaris

Characterized as a chronic inflammatory condition of the pilosebaceous unit, acne vulgaris primarily affects adolescents and young adults, although its clinical course often extends into adulthood [71,72]. Although the current therapeutic armamentarium, which includes topical retinoids, antimicrobial agents, and systemic isotretinoin, remains the gold standard [73], its utility is often limited by significant skin irritation and the need for prolonged daily treatment regimens. These factors often compromise patient adherence, leading to suboptimal clinical outcomes. PDT represents a promising alternative to conventional strategies, utilizing specific wavelengths of light to selectively target sebaceous glands and modulate the local inflammatory environment. Also, PDT exerts a multifaceted influence on the immune microenvironment within the pilosebaceous unit, thereby significantly reducing the overall burden of lesions [74]. A comprehensive systematic review involving over 4300 patients across 82 studies confirms the efficacy of this approach, particularly when using protocols based on 5-ALA or MAL [75]. The summarized data demonstrate consistent clinical improvement in inflammatory lesions and a marked reduction in glandular hyperactivity, resulting in high levels of patient satisfaction. Although complete remission remains a difficult endpoint to achieve in most cohorts, the safety profile of PDT is remarkably favorable: adverse events are generally transient and self-limiting and manifest as mild erythema, desquamation, or localized discomfort. However, the marked heterogeneity of administration parameters (including photosensitizer concentration, incubation duration, and light dosimetry) underscores the critical need for protocol standardization and rigorous head-to-head comparisons with established conventional standards. Research in the adolescent population is of clinical interest, as treatment options for this group are often limited by safety concerns regarding systemic agents. A recent case report highlighted the successful management of a 16-year-old with severe, recalcitrant nodular acne of the trunk, which had proven refractory to both oral minocycline and isotretinoin. Treatment consisted of five monthly sessions of topical application of 10% 5-ALA for three hours, followed by irradiation with 630 nm red light at a fluence of 75 J/cm^2^ [76]. A marked clinical improvement was observed, with resolution of nodules and pustules, relief of pain, and a significant esthetic improvement, particularly in the sternal region. The procedure was well tolerated, with transient pain and erythema during the initial sessions and no long-term adverse effects. This case further confirms the potential of PDT as a safe and effective treatment option for adolescents with refractory acne, particularly when conventional therapies fail or are contraindicated.

#### 2.2.2. Infectious Dermatology

PDT has progressively expanded beyond its traditional oncologic indications into infectious dermatology, where its antimicrobial activity is mediated by ROS-induced cellular damage combined with local immunomodulatory effects. Beyond direct cytotoxicity toward microorganisms and infected keratinocytes, PDT enhances antigen presentation, cytokine release, and recruitment of inflammatory cells, promoting pathogen clearance while preserving surrounding tissue integrity. These characteristics allow selective targeting of infected structures with excellent cosmetic outcomes and compatibility with delicate anatomical sites. Although the level of evidence varies among diseases, the current literature supports a therapeutic role across viral, fungal, parasitic, and bacterial infections [77] (Table 2 and Table 3).

The most substantial body of clinical evidence regarding the antimicrobial applications of PDT pertains to HPV-related pathologies, including common, plantar, and periungual warts, as well as anogenital condylomata and epidermodysplasia verruciformis (EV). The therapeutic selectivity in these conditions involves the preferential accumulation of photosensitizers within HPV-dysregulated keratinocytes, which exhibit heightened vulnerability to oxidative stress due to altered differentiation and attenuated antioxidant defenses. Clinical clearance is consistently optimized in recalcitrant or hyperkeratotic presentations when preparatory measures, such as curettage or keratolytics, are employed to facilitate deeper photosensitizer diffusion. This inherently tissue-sparing modality is particularly valuable for periungual lesions, where it safeguards against the permanent nail matrix injury often associated with destructive procedures [78,79,80,81,82]. In the management of condylomata acuminata, PDT offers a sophisticated alternative to ablative methods, providing selective cytotoxicity with high tolerability on mucosal surfaces [83,84,85,86,87,88,89,90]. These benefits extend significantly to the pediatric population, for whom conventional therapies are often poorly tolerated or prone to recurrence. Furthermore, in the context of EV, targeted PDT can reduce cumulative morbidity by allowing for the simultaneous control of multiple lesions [91,92,93]. In addition to HPV-mediated diseases, molluscum contagiosum (particularly in pediatric or immunocompromised cohorts) responds favorably through localized cytotoxicity and immune activation [94,95,96], while preliminary investigations into herpes simplex virus (HSV) suggest accelerated resolution of lesions and reduced recurrence rates, especially in chronic or antiviral-refractory forms [97,98,99]. PDT is attracting growing interest as an additional or alternative therapeutic option when antifungal treatment fails, is contraindicated, or is poorly tolerated. ROS-mediated damage destroys the cell walls, membranes, and intracellular components of fungi, mechanisms that are less prone to the development of resistance compared to conventional antifungal agents [100,101,102,103]. Onychomycosis is the most extensively studied fungal indication (EDF Level B). The ability of PDT to penetrate hyperkeratotic nail plates and destroy fungal biofilms is a key advantage. Pre-treatment strategies such as chemical keratolysis, microperforation of the nail plate, or fractional CO_2_ laser improve drug delivery and efficacy, with response rates ranging from 30% to 70% and excellent tolerability [100,101,102,103,104,105,106,107,108,109,110,111]. In superficial mycoses, including dermatophytosis, candidiasis, and Malassezia-associated dermatoses, small clinical studies demonstrate a reduction in erythema, desquamation, and fungal burden, particularly in recurrent or treatment-resistant diseases [111]. In deeper infections such as chromoblastomycosis, sporotrichosis, and phaeoidomycosis, PDT primarily acts as an adjunct to systemic antifungals, accelerating healing and shortening the duration of treatment. Pigmented fungi appear particularly sensitive due to their increased light absorption [112,113,114]. Furthermore, PDT has established itself as a non-invasive and cosmetically advantageous modality for the treatment of cutaneous leishmaniasis (CL), a role currently supported by level B evidence according to the EDF. The therapeutic rationale is based on a multi-pronged approach: direct parasiticidal activity mediated by ROS against intracellular amastigotes, a favorable immunomodulatory shift characterized by Th1 polarization, and the resulting stimulation of tissue repair [115,116]. Clinical studies using ALA or MAL in combination with red light irradiation have documented significant flattening of lesions and re-epithelialization, consistently achieving parasitological cure with minimal scarring. These results make PDT particularly valuable for patients intolerant to systemic antimonials, as well as for those with ulcerative, chronic, or cosmetically sensitive facial lesions. Furthermore, it serves as a powerful component of multimodal regimens, demonstrating notable synergy with antimonials, cryotherapy, or thermotherapy [117,118,119]. Although species-specific variations exist, with the strongest evidence currently available for L. major and L. tropica, PDT remains a sophisticated, low-toxicity therapeutic option in this field. In the field of bacterial infections, PDT offers a broad-spectrum antimicrobial profile that encompasses Gram-positive, Gram-negative, and antibiotic-resistant organisms, with the key advantage of simultaneously acting on protective biofilms. Given its non-specific oxidative mechanism, the development of microbial resistance is considered highly unlikely [120,121,122,123,124]. Clinical observations on impetigo caused by S. aureus and S. pyogenes show a rapid reduction in bacterial load and scab formation, accompanied by accelerated healing times, advantages that are particularly relevant in cases of drug resistance or where cosmetic outcomes are a priority [125]. Similarly, ALA-PDT has been shown to alleviate pustules and inflammatory papules in chronic bacterial folliculitis, with documented benefits even in refractory cases involving Gram-negative bacteria or Pseudomonas [126]. Perhaps one of the most significant applications of this technology is its efficacy against MRSA; PDT not only eradicates resistant strains but also disrupts the biofilm architecture and acts synergistically with systemic antibiotics in the management of infected ulcers, recurrent abscesses, and chronic wound colonization [127,128]. In fact, the ability to destroy the biofilm represents an important clinical strength, particularly in the treatment of chronic venous, diabetic, and pressure ulcers. In these contexts, the PDT-mediated reduction in exudate, odor, and bacterial load facilitates better healing and restores the wound microenvironment’s responsiveness to standard care [129].

### 2.3. Photorejuvenation and Cosmetic Use of PDT

In addition to its well-established use in oncology, PDT has emerged as a powerful non-ablative modality for skin photorejuvenation. The procedure, which involves the topical application of ALA or MAL followed by targeted light activation, triggers the generation of ROS to induce controlled epidermal stress. This process subsequently orchestrates profound dermal remodeling, characterized by robust neocollagenesis and complete renewal of the extracellular matrix [130,131,132,133]. From a clinical perspective, the efficacy of PDT is evident in the significant improvement of wrinkles, discoloration, texture irregularities, and pore visibility: results achieved with minimal recovery time for the patient and a reassuring safety profile. The therapeutic impact is often amplified by synergistic strategies, such as microneedling, laser-assisted drug delivery, or fractional CO_2_ pretreatment, all of which serve to improve the bioavailability of the photosensitizer and stimulate deeper collagen synthesis [99,134,135,136]. Although the therapeutic parameters reported in the literature remain inconsistent, achieving consistent esthetic results is intrinsically linked to the optimization of light fluence, treatment frequency, and photosensitizer incubation times. Ultimately, PDT is a versatile and minimally invasive procedure that offers significant aesthetic improvement in the management of skin affected by chronic photodamage (Table 4).

### 2.4. Emerging and Next-Generation Photosensitizers in Dermatologic Photodynamic Therapy

Beyond the classical distinction between porphyrin derivatives and newer synthetic compounds, the evolution of PDT has progressively led from first- and second-generation photosensitizers to third-generation systems, which are designed to improve tumor selectivity, pharmacokinetics, safety, and theranostic potential. In particular, third-generation photosensitizers are typically obtained by conjugating established second-generation agents with tumor-targeting moieties, such as antibodies, peptides, amino acids, or carbohydrates, or by encapsulating them into advanced delivery platforms including liposomes, micelles, and nanoparticles [137,138]. These approaches aim to increase bioavailability, prolong circulation time, improve solubility and stability, and enhance preferential accumulation within diseased tissue while reducing off-target phototoxicity. In addition, theranostic designs incorporating imaging probes allow real-time monitoring of biodistribution and treatment response, whereas linker engineering can further optimize hydrophilic/hydrophobic balance and cellular uptake [139]. Overall, these innovations support a more precise and mechanism-oriented use of PDT and may help overcome resistance-related limitations that still constrain conventional photosensitizers. Within this broader framework, porphyrin-related compounds, particularly chlorins, remain highly relevant for dermatologic PDT because their absorption in the red spectrum allows deeper tissue penetration while preserving strong photodynamic activity [140]. Chlorin e6 has shown encouraging results in basal cell carcinoma (BCC). In a case report of a 78-year-old man with nodular nasal BCC, intravenous chlorin e6 at 0.08 mg/kg followed, after 3 h, by 665 nm laser irradiation at 150 J/cm^2^ achieved complete lesion clearance after a single treatment session, without reported adverse effects during a 2-month follow-up [141]. More importantly, a larger 2025 clinical series including 1782 patients with BCC treated with intravenous Fotoran E6^®^ (chlorin e6, 1.0 mg/kg), 2 h drug-light interval, and 670 nm laser irradiation reported complete tumor regression at 1 month, with recurrence rates of 1% at 1 year and 8.5% over 5 years, together with excellent cosmetic outcomes [142]. These findings support the view that chlorin-based PDT may represent a safe, minimally invasive, and cosmetically advantageous option for selected cutaneous tumors, particularly when tissue preservation is a priority. Therefore, cyanine dyes represent another promising class of photosensitizers for skin oncology [140]. Their main advantage is that their absorption maxima lie in the NIR-I region, enabling deeper tissue penetration than many conventional agents; moreover, some compounds, including indocyanine green (ICG) and IRDye800-related systems, can also generate off peak emission in the NIR-II window (1000–1700 nm), which may improve tumor visualization and image contrast. ICG is already FDA-approved as a diagnostic dye, while heptamethine cyanines are of particular interest because they show preferential uptake by cancer cells, partly mediated by organic anion-transporting polypeptides, which are often overexpressed in tumors [142,143]. However, cyanines still present relevant limitations, especially poor water solubility and relatively low ROS generation; for this reason, structural modifications, halogenation, metal incorporation, and nanoformulation strategies are being explored to enhance phototoxicity, tumor selectivity, and clinical applicability in PDT.

## 3. Discussion

**Figure 2:**
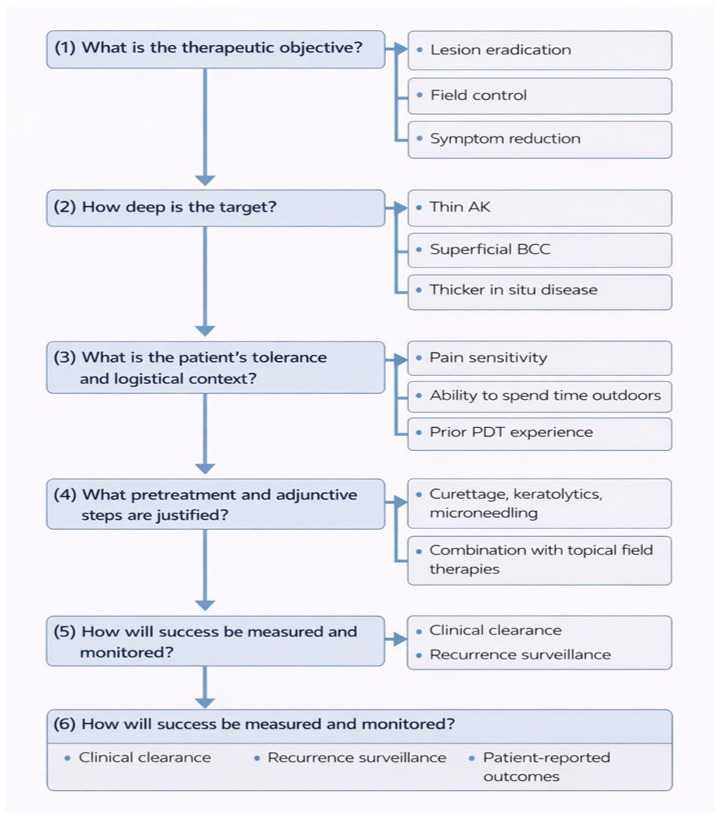
Mechanism-oriented framework for practical PDT protocol selection. The algorithm guides clinicians through sequential questions addressing therapeutic objective (lesion eradication, field control, symptom reduction), target depth (e.g., thin actinic keratosis, superficial basal cell carcinoma, thicker in situ disease), patient tolerance and logistics (pain sensitivity, feasibility of daylight exposure, prior PDT experience), justified pretreatment and combination strategies (curettage, keratolytics, microneedling, adjunct topical therapies), and outcome assessment. Treatment success should be evaluated using a multidimensional approach including clinical clearance, recurrence surveillance, and patient-reported outcomes.

PDT has developed from a restricted procedure based on selective premalignant lesions, to a versatile therapeutic platform that is applicable in oncologic, inflammatory, infectious, and esthetic dermatology. Furthermore, PDT is not only cytotoxic by reactive oxygen species, but also in the multimodal biological activity of vascular shutdown, microbiological disruption, and immune modulation. Rather than acting as a standard “destructive” therapy, PDT is increasingly being identified as a biologically active field therapy that can remodel the cutaneous microenvironment. Nevertheless, the diversity of protocols, indications, and endpoints noted across this review would suggest that contemporary clinical guidelines do not reflect the present. Moreover, there is considerable variability in the strength of clinical evidence across different indications. PDT efficacy is well supported by robust clinical trials in conditions such as basal cell carcinoma, actinic keratosis, and Bowen’s disease, whereas the evidence for other applications, including Kaposi’s sarcoma, cutaneous lymphomas, and infectious diseases, remains limited. In these cases, the available data are largely derived from case reports and small case series, which precludes the formulation of definitive or broadly generalizable conclusions. Many guidelines maintain classification of PDT by disease labels (AK, BCC, acne, infections); however, there is evidence that treatment success relies more on tissue characteristics (epidermal vs. dermal target, microbial vs. neoplastic burden, inflammatory component, depth of involvement) as well as patient-related factors, i.e., tolerability, adherence, immunosuppression, and recurrence risk. Pain is one of the major practical barriers to wider use, especially in case of extensive field cancerization of the facial or scalp regions. Thus, protocol engineering, namely, through daylight or simulated daylight activation, shorter incubation with instant illumination, lower irradiance or fractionated illumination, and supportive cooling, is a logical optimization approach and not a simple comfort adjustment (Figure 2).

Hence, future guidelines should progressively move beyond a purely disease-centered positioning of PDT and adopt more mechanism-oriented algorithms, clarifying when PDT should be used primarily as a cytotoxic treatment, a field-directed immunomodulatory strategy, an antimicrobial approach, or a tissue-remodeling stimulus. In this perspective, PDT is increasingly unlikely to remain a stand-alone intervention and should instead be framed within combination and sequential treatment strategies. Dermatologic therapy is becoming more multimodal, and in both keratinocyte carcinogenesis and field cancerization, procedural-topical regimens are gaining relevance for long-term disease control. Recent short-course topical agents, such as tirbanibulin and 5-fluorouracil, represent an important advance in this context: their antiproliferative activity and manageable tolerability profiles may complement the cytotoxic and immunogenic effects of PDT, enabling staged or maintenance strategies aimed at reducing recurrence while limiting cumulative irritation [144]. A similar synergistic principle is already being applied in other contexts, including the combination of PDT with fractional lasers for non-melanoma skin cancer, with antifungals or antibiotics for infections, and with microneedling or other energy-based devices in photorejuvenation. Therefore, the future of PDT appears less focused on competition and more on synergy, with PDT serving as a supportive and adaptable component within broader therapeutic pathways. This integrative role is likely to expand further thanks to technological innovation. Considering the limitations of PDT, one of the major is still the shallow penetration depth of activating light, which restricts efficacy in deeper tumors. Future developments may address this issue through longer-wavelength activation systems, more powerful or alternative energy sources, and even X-ray-activated strategies, all of which aim to improve tissue penetration and expand the therapeutic range of PDT for dermal tumors, vascular lesions, and refractory infections [145]. At the same time, advances in photosensitizer chemistry and drug delivery could redefine the performance of PDT. New photosensitizers, including polymer-based and quantum dot-based systems, have demonstrated enhanced photosensitivity and biocompatibility, while nanotechnology-based delivery platforms could improve selective accumulation at target sites and reduce collateral damage to surrounding healthy tissues [146,147]. Nanoparticles are particularly promising not only as carriers for photosensitizers but also as multifunctional platforms capable of simultaneously delivering additional agents, including immunomodulators and chemotherapeutic agents, thereby enhancing the biological effects of PDT [148]. In this regard, strategies combining PDT with chemotherapy, radiation therapy, immunotherapy, and photothermal therapy are particularly promising, as a growing body of evidence suggests significant synergistic interactions capable of enhancing tumor destruction and overall treatment efficacy [147]. Experimental nanoplatforms that co-encapsulate 5-ALA and doxorubicin, for example, suggest that PDT may evolve toward theranostic systems that integrate treatment, targeting, and real-time diagnostic capabilities within a single platform [149]. Another important future direction is the integration of imaging tools and objective monitoring into PDT workflows. Non-invasive imaging techniques could improve patient selection, detect disease at the subclinical stage, define treatment margins more precisely, and optimize the timing of retreatment, thereby transforming PDT from an episodic intervention into a more dynamic and personalized management strategy [150,151]. Concurrently, the development of biomarkers predictive of PDT response, tolerability, and recurrence risk could enable better patient stratification and more rational protocol selection. This precision-based approach is particularly relevant as dermatology increasingly converges with personalized medicine. From a broader perspective, PDT also represents a compelling model for sustainable dermatology. Also, its mechanism does not promote microbial resistance, is minimally invasive to tissues, and, in selected contexts, can reduce the need for prolonged systemic therapy. These characteristics are particularly relevant in an era of growing concern regarding antimicrobial stewardship, chronic inflammation, and therapeutic burden.

## 4. Materials and Methods

A literature search was conducted in PubMed/MEDLINE and Scopus to identify relevant evidence on the mechanisms, protocols, and dermatologic applications of photodynamic therapy. Owing to the narrative design of this review, study selection followed a pragmatic approach based on clinical relevance and quality of evidence. Detailed search criteria and the study selection flowchart are reported in Supplementary File.

## 5. Conclusions

In conclusion, the future of photodynamic therapy in dermatology will depend less on expanding its indications and more on refining its role within integrated treatment pathways. Future efforts should focus on protocol standardization, identification of predictors of response, and the development of combination and sequential strategies with other medical, surgical, and technological approaches. Updated guidelines should also incorporate objective outcome measures and patient-centered endpoints, moving beyond purely diagnostic classifications toward a more mechanistic and pragmatic use of PDT. In this perspective, PDT is likely to evolve from a lesion-directed procedure into a flexible therapeutic platform for long-term dermatologic management.

## Figures and Tables

**Table 1:** Comparative clinical positioning of photodynamic therapy (PDT) in actinic keratosis (AK), basal cell carcinoma (BCC), and squamous cell carcinoma (SCC).

ASPECT	Actinic Keratosis (AK)	Basal Cell Carcinoma (BCC)	Squamous Cell Carcinoma (SCC)
Disease Type	Premalignant lesion	Low-grade malignant tumor	Malignant tumor with metastatic potential
Typical Clinical Setting	Multiple lesions; field cancerization	Small, low-risk tumors in cosmetically sensitive areas	Superficial or microinvasive lesions only
Risk of Progression/Metastasis	Progression to SCC (up to 16%/year)	Very low metastatic risk	Significant metastatic risk
Role of PDT	First-line field therapy	Alternative to surgery in selected cases	Not recommended as standard monotherapy
Main Indications	Olsen grade I–II AK; extensive fields	Superficial BCC; selected nodular BCC	Superficial or microinvasive SCC; adjunctive use
Efficacy (Short-Term)	Complete response ≈ 90%	sBCC: CR 80–97%	CR 54–100% (superficial lesions only)
Long-Term Control/Recurrence	Good control with retreatment	Recurrence 6–22% (higher in difficult cases)	High recurrence (~69%)
Comparison with Surgery	Comparable to cryotherapy or 5-FU	Slightly inferior to surgery	Inferior oncologic control
Combination Strategies	cPDT, dlPDT, adlPDT	Debulking, curettage before PDT	Fractional laser-assisted PDT
Cosmetic Outcome	Excellent (96–98%)	Excellent/good (82–95%)	Excellent in selected cases
Tolerability	Mild–moderate pain	Moderate pain; may require repeated sessions	Pain and longer treatment protocols
Main Limitations	Reduced efficacy in thick lesions	Incubation time; recurrence risk	High recurrence rate; limited evidence
Therapeutic Positioning	Optimal indication for PDT	Valid alternative for selected low-risk tumors	Adjunctive or investigational role

**Table 2:** Evidence Levels for PDT in Infectious Diseases.

Condition	Pathogen Category	Evidence Level (EDF 2019 + Recent Literature)	Notes
Common warts plantar warts	Viral (HPV)	B	Best evidence among viral diseases; enhanced by keratolytics.
Periungual warts	Viral (HPV)	B	High recurrence reduction; tissue-sparing.
Condylomata acuminata	Viral (HPV)	B	Strong adult data; ideal for pediatric sensitive areas.
Epidermodysplasia verruciformis	Viral	C	Useful as adjunct; long-term disease control.
Molluscum contagiosum	Viral (poxvirus)	C	Pediatric-friendly, non-invasive.
HSV infections	Viral (HSV-1, HSV-2)	C	Adjunct role; potential recurrence reduction.
Onychomycosis	Fungal	B	Best fungal indication; enhanced with pretreatment.
Superficial fungal infections	Fungal	C	Useful in recurrent or resistant cases.
Deep cutaneous mycoses	Fungal	C	Chromoblastomycosis shows best responses.
Cutaneous leishmaniasis	Protozoan	B	Excellent cosmetic results; high response rates in L. major/tropica.
Impetigo/pyodermas	Bacterial	C	Useful in resistant cases and facial disease.
Folliculitis	Bacterial	C	Particularly helpful in chronic cases.
Acne (C. acnes)	Bacterial-associated	B	Strong evidence for inflammatory acne.
MRSA infections	Bacterial	C	Effective against resistant strains; strong in vitro evidence.
Biofilm-associated chronic ulcers	Mixed bacteria	C	Biofilm disruption improves healing.

**Table 3:** Typical PDT Parameters in Infectious Diseases.

Infection Type	Photosensitizer	Incubation Time	Light Source	Fluence	Frequency
Viral warts	ALA/MAL	1–3 h	Red LED 630–635 nm	37–100 J/cm^2^	Every 1–2 weeks, 2–4 sessions
Condyloma	ALA	2–3 h	Red LED	60–100 J/cm^2^	Weekly × 2–4
Molluscum	ALA	1–2 h	Red or blue light	10–100 J/cm^2^	1–3 sessions
HSV	ALA + antiviral adjunct	1–2 h	Red	37–75 J/cm^2^	During prodrome/outbreak
Onychomycosis	ALA/MAL + pretreatment	3–4 h	Red	37–100 J/cm^2^	Weekly or biweekly, 3–6 sessions
Superficial mycoses	ALA/MAL	1–3 h	Red	37–100 J/cm^2^	Weekly, 1–3 sessions
Deep mycoses	ALA	3 h	Red	75–100 J/cm^2^	Weekly × 4–8
Cutaneous leishmaniasis	ALA/MAL	3 h	Red	37–100 J/cm^2^	Weekly or biweekly × 1–4
Impetigo/pyoderma	ALA	1–2 h	Red/blue	10–75 J/cm^2^	1–3 sessions
Folliculitis	ALA	1–3 h	Red	37–100 J/cm^2^	2–4 sessions
Acne	ALA	1–3 h	Blue + red combination	10–100 J/cm^2^	Every 2–4 weeks × 1–3

**Table 4:** PDT parameters for skin photorejuvenation.

Treatment Modality	Photosensitizer	Incubation Time	Light Source	Wavelength	Fluence	Sessions	Notes/Evidence
Conventional ALA-PDT	20% 5-ALA	1–3 h	Red LED	630–635 nm	20–80 J/cm^2^	1–3	Improves texture, pigmentation, fine lines; stronger rejuvenation but more erythema.
Conventional MAL-PDT	MAL cream 16%	30–180 min	Red LED	630–635 nm	37–75 J/cm^2^	1–3	Better tolerated than ALA; used with field treatments for actinic damage + rejuvenation.
Daylight-PDT (DPDT)	5-ALA nano-emulsion	30 min	Natural daylight	Broad spectrum	N/A (continuous low-dose activation)	1–2	Minimal pain; reduces roughness and lentigines; ideal for sensitive patients.
PDT + Fractional Co_2_	ALA or MAL	30–60 min	Red LED	630 nm	20–40 J/cm^2^	1–2	Fractional CO_2_ enhances penetration; significantly increases neocollagenesis.
PDT + Microneedling	ALA (10–20%)	30–90 min	Red LED	630 nm	20–40 J/cm^2^	2–3	Split-face studies show superior wrinkle reduction vs. PDT alone.
PDT + IPL (Photorejuvenation-Pdt)	ALA (5–20%)	Short-contact 30–60 min	IPL (500–1200 nm)	Broad	According to IPL settings	1–3	Useful for lentigines, redness; synergistic epidermal turnover.
Laser-Assisted PDT	ALA/MAL	10–30 min	Red LED	630 nm	20–60 J/cm^2^		

## Data Availability

No new data were created or analyzed in this study. Data sharing is not applicable to this article.

## Supplementary Materials

The following supporting information can be downloaded at: https://www.mdpi.com/article/10.3390/ijms27093960/s1.

## Abbreviations

The following abbreviations are used in this manuscript:

PDT	Photodynamic therapy
5-ALA	5-aminolevulinic acid
PS	photosensitizer
ROS	reactive oxygen species
cPDT	conventional PDT
dlPDT	daylight PDT
adlPDT	artificial daylight PDT
LED	light-emitting diode
MAL	methyl aminolevulinate
PpIX	protoporphyrin IX
AK	actinic keratosis
SCC	squamous cell carcinoma
BCC	basal cell carcinoma
5-FU	5-fluorouracil
BD	Bowen’s disease
mTHPC	Temoporfin
PCL	Primary Cutaneous Lymphoma
LyP	lymphomatoid papulosis
MF	mycosis fungoides
CBCL	cutaneous B-cell lymphomas
KS	Kaposi’s sarcoma
HHV-8	Human Herpesvirus 8
HIV	Human Immunodeficiency virus
ABCG2	ATP-binding cassette super-family G member 2
ISPI	in situ photoimmunotherapy
HPV	Human Papillomavirus
RL	Red light
BL	Blue Light
