# Supplementary material for: Photodynamic Therapy in Dermatology
Source: Int J Mol Sci. 2026 Apr 29;27(9):3960. doi: 10.3390/ijms27093960 (PMC13163354; doi:10.3390/ijms27093960)
Supplement: Supplementary file 1 [file ijms-27-03960-s001.zip › ijms-4252076-supplementary.pdf]

Databases and search period

A comprehensive literature search was performed in PubMed/MEDLINE and Scopus to identify publications relevant to the mechanisms, protocols, and dermatologic applications of photodynamic therapy (PDT). The search covered all records available in each database from inception to March 31, 2026. No restrictions were applied regarding language, publication date, or study design at the initial search stage.

Search strategy

The search strategy combined terms related to photodynamic therapy, photosensitizers, light sources, and dermatologic indications. The following keywords and their variants were used:

("photodynamic therapy" OR "PDT" OR "aminolevulinic acid" OR "5-ALA" OR "methyl aminolevulinate" OR "MAL" OR "protoporphyrin IX" OR "PpIX" OR "daylight photodynamic therapy" OR "dlPDT" OR "artificial daylight PDT" OR "light-emitting diode" OR "LED") AND ("dermatology" OR "skin" OR "actinic keratosis" OR "field cancerization" OR "Bowen disease" OR "squamous cell carcinoma" OR "basal cell carcinoma" OR "nonmelanoma skin cancer" OR "acne" OR "photoaging" OR "photorejuvenation" OR "warts" OR "HPV" OR "molluscum contagiosum" OR "onychomycosis" OR "cutaneous leishmaniasis" OR "cutaneous lymphoma" OR "mycosis fungoides" OR "Kaposi sarcoma" OR "Paget disease")

Where appropriate, database-specific syntax and controlled vocabulary were adapted to improve sensitivity.

Eligibility criteria

Inclusion criteria

Studies were considered eligible if they met one or more of the following criteria:

- addressed the mechanisms of action of PDT, including photochemistry, light-tissue interaction, immunologic effects, vascular effects, or pharmacology of photosensitizers;
- reported on clinical applications of PDT in dermatology, including oncologic, inflammatory, infectious, and cosmetic indications;
- evaluated treatment protocols, including conventional PDT, daylight PDT, artificial daylight PDT, or protocol modifications;
- were original peer-reviewed studies, including randomized controlled trials, prospective or retrospective observational studies, case series, or clinically relevant translational/preclinical investigations;
- were systematic reviews, meta-analyses, guidelines, or consensus documents relevant to the topic.

Narrative reviews and expert opinions were also considered when useful to contextualize areas in which evidence was limited, heterogeneous, or emerging.

Exclusion criteria

Studies were excluded if they:

- did not concern photodynamic therapy;
- did not concern dermatologic or cutaneous indications;
- were unrelated to the scope of the review (for example, exclusively non-dermatologic applications without relevant translational implications for skin disease);
- lacked sufficient methodological or clinical relevance;
- were duplicate records.

Study selection

After removal of duplicates, titles and abstracts were screened for relevance. Full texts were then assessed when necessary to confirm eligibility. Study selection followed a pragmatic approach consistent with the narrative nature of the review, emphasizing clinical relevance, methodological quality, and representativeness of the available evidence across the main dermatologic indications and emerging applications of PDT.

Priority was given to:

1. randomized controlled trials, when available;
2. prospective and retrospective clinical studies;
3. systematic reviews and meta-analyses;
4. guidelines and consensus statements;
5. mechanistic, translational, and preclinical studies with clear dermatologic relevance.

Data synthesis approach

Because of the narrative design of this review and the heterogeneity of the included literature, no formal quantitative synthesis was planned. The selected studies were synthesized descriptively according to major thematic areas, including:

- mechanistic basis of PDT;
- photosensitizers and illumination strategies;
- neoplastic and premalignant indications;
- inflammatory and infectious indications;
- cosmetic and photorejuvenation applications;
- emerging technologies and future directions.

Flowchart of study selection

The study selection process is summarized in the accompanying flowchart and includes the following phases (Figure S1):

1. Identification of records through database searching;
2. Deduplication of retrieved records;
3. Screening of titles and abstracts;
4. Eligibility assessment of full-text articles;
5. Inclusion of studies in the qualitative narrative synthesis.

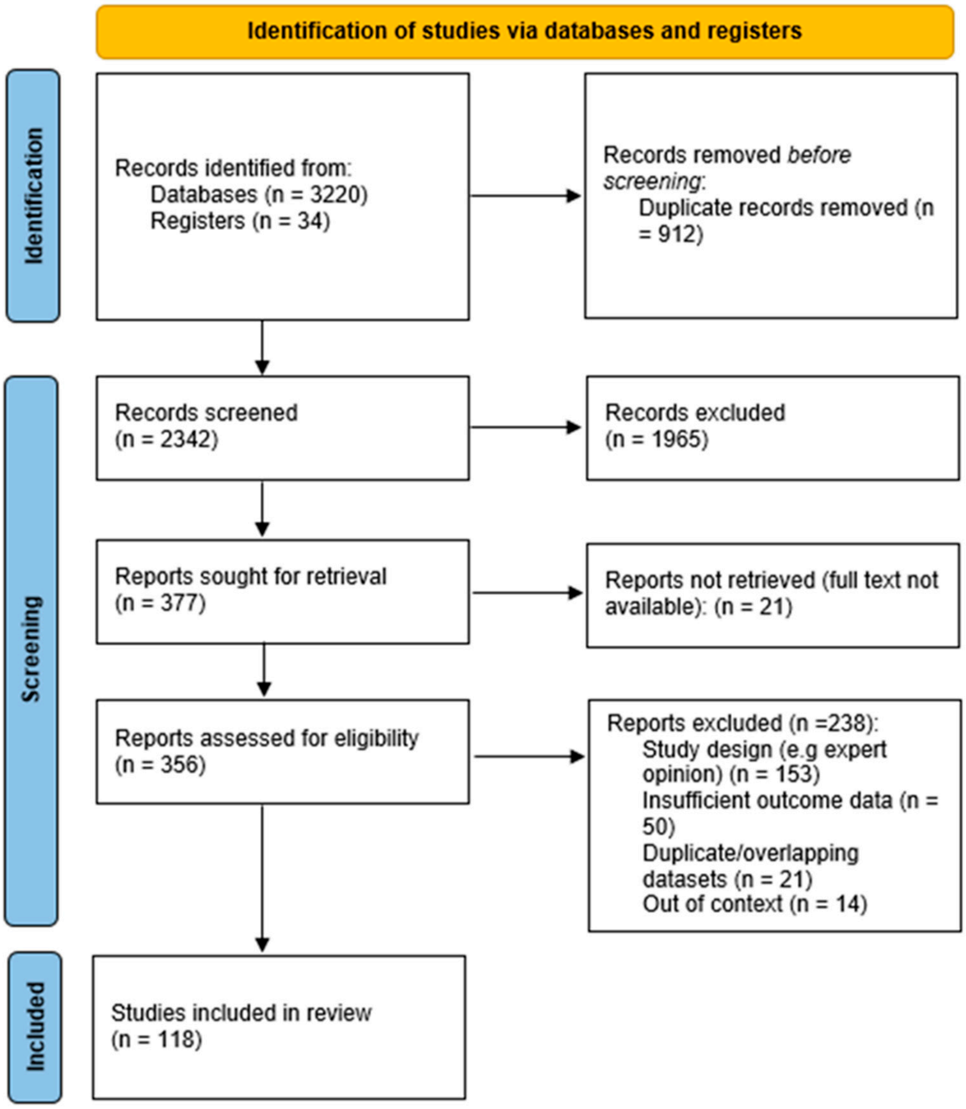

Figure S1. Study selection diagram. Flow of records identified, deduplicated, screened (title/abstract), assessed in full text, and included in the qualitative synthesis of this review on PDT.
